# Gender Differences in Post-Operative Human Skin

**DOI:** 10.3390/biomedicines11102653

**Published:** 2023-09-27

**Authors:** Barbara Gawronska-Kozak, Marta Kopcewicz, Sylwia Machcinska-Zielinska, Katarzyna Walendzik, Joanna Wisniewska, Justyna Drukała, Tomasz Wasniewski, Joanna Rutkowska, Piotr Malinowski, Michał Pulinski

**Affiliations:** 1 Institute of Animal Reproduction and Food Research, Polish Academy of Sciences, 10-748 Olsztyn, Poland; m.kopcewicz@pan.olsztyn.pl (M.K.); s.machcinska@pan.olsztyn.pl (S.M.-Z.); k.walendzik@pan.olsztyn.pl (K.W.); j.bukowska@pan.olsztyn.pl (J.W.); 2 Department of Cell Biology, Faculty of Biochemistry, Biophysics and Biotechnology, Jagiellonian University, 31-007 Krakow, Poland; justyna.drukala@uj.edu.pl; 3 Department of Obstetrics, Perinatology and Gynecology, School of Medicine, Collegium Medicum, University of Warmia and Mazury in Olsztyn, 10-719 Olsztyn, Poland; tomasz.wasniewski@uwm.edu.pl; 4 Department of Internal Medicine, Clinic of Endocrinology, Diabetology and Internal Medicine, School of Medicine, Collegium Medicum, University of Warmia and Mazury in Olsztyn, 10-719 Olsztyn, Poland; joanna.rutkowska@uwm.edu.pl; 5 Department of Surgery, School of Medicine, Collegium Medicum, University of Warmia and Mazury in Olsztyn, 10-719 Olsztyn, Poland

**Keywords:** skin, scar, human, gender, dWAT, ECM, FOXN1

## Abstract

Although the impact of age, gender, and obesity on the skin wound healing process has been extensively studied, the data related to gender differences in aspects of skin scarring are limited. The present study performed on abdominal human intact and scar skin focused on determining gender differences in extracellular matrix (ECM) composition, dermal white adipose tissue (dWAT) accumulation, and Foxn1 expression as a part of the skin response to injury. Scar skin of men showed highly increased levels of *COLLAGEN 1A1*, *COLLAGEN 6A3*, and *ELASTIN* mRNA expression, the accumulation of thick collagen I-positive fibers, and the accumulation of α-SMA-positive cells in comparison to the scar skin of women. However, post-injured skin of women displayed an increase (in comparison to post-injured men’s skin) in collagen III accumulation in the scar area. On the contrary, women’s skin samples showed a tendency towards higher levels of adipogenic-related genes (*PPARγ*, *FABP4*, *LEPTIN*) than men, regardless of intact or scar skin. Intact skin of women showed six times higher levels of *LEPTIN* mRNA expression in comparison to men intact (*p* < 0.05), men post-injured (*p* < 0.05), or women post-injured scar (*p* < 0.05) skin. Higher levels of FOXN1 mRNA and protein were also detected in women than in men’s skin. In conclusion, the present data confirm and extend (dWAT layer) the data related to the presence of differences between men and women in the skin, particularly in scar tissues, which may contribute to the more effective and gender-tailored improvement of skin care interventions.

## 1. Introduction

Skin, the largest organ of the body, fulfills barrier and sensory functions, as classical studies have shown, but also, as recent data revealed, is an integral part of the endocrine, nervous, and immune systems [1]. Consequently, the impairment in internal organ performance very often manifests in aberrant skin appearance, which is the first signal of dysfunction or disease [2]. 

Skin is composed of two well-defined and separated basement membrane layers: the upper epidermis and the lower dermis. The dermis is a heterogeneous layer that consists of papillary and reticular layers and recently distinguished dermal white adipose tissue (dWAT) [3]. Papillary and reticular fibroblasts, although they arise from distinct embryonic lineages and fulfill different functional assignments in maintaining skin homeostasis, share the ability to synthesize and maintain extracellular matrix (ECM) [4]. The ECM forms the architectural structure of the skin through its components: collagen (types I, III, IV, V, VI, VII, XII, XIII, XIV, XVII, XXIII), elastic fibers (elastin, fibrillin-1 and -2), glycoproteins, proteoglycans, and galactosaminoglycans/glycosaminoglycans [5]. But it also actively supports the interplay between cellular components and the microenvironment to regulate skin homeostasis and response to wounding [5]. The dWAT layer has recently gained a lot of attention due to its contribution to wound healing [6,7,8], thermoregulation [9], hair follicle growth [10,11,12], and immune response [13,14]. Experiments based on animal/mouse models showed that in post-injured skin, dWAT participates in fibroblast recruitment, stimulates macrophage infiltration, and enhances immune defense [6,7,8].

Skin, as a first line of the environmental-to-internal organ barrier, is vulnerable to disruption of its integrity due to injuries or surgical interventions. The healing of injured skin completes as a collagenous scar, which recovers skin continuity but impairs sensory, endocrine, nervous, thermoregulatory, and immune functions. Scar is beneficial due to the patch of disrupted skin continuity. However, accumulation of collagen excess, lack of dermal appendages, and attenuation in stimuli reception in the scar impair skin performance, which may cause disabilities and psychological burden. 

The wound healing process, scar size, and post-wounding skin quality depend on injury type (i.e., burn vs. incision), injury size, location of the wound on the body region, individual predispositions, and age [15,16]. The analysis of the skin wound healing process as a function of age, sex, and diet revealed that age as a single variable has the most fundamental impact [17]. A study by Ashcroft et al. (2002) showed that the healing of skin injuries at an older age takes longer but is portrayed as scarless skin healing, similar to that observed in mammalian fetuses [18].

However, the combination of two factors—age and sex—had the most profound effect on skin and skin wound healing [17]. Older C57Bl/6 male mice displayed a sharp reduction in collagen I and III expression in intact skin but also during the skin wound healing process. Age and sex also impacted matrix metalloproteinase 13 (Mmp-13) expression, which was the highest for young C57Bl/6 female mice at days 3 and 7 after injury. Interestingly, physiologically related to old age, a decrease in skin thickness was compensated by an increase in dWAT thickness for high-fat diet (HFD)-fed older mice, although gender differences were not examined in these studies [17]. 

Cutaneous wound healing and, as a consequence, scar formation are regulated by a broad spectrum of transcription factors (AP-1, Pparβ/δ, HoxA3, HoxD3), growth factors (TGFβ, PDGF, FGF), cytokines (i.e., Il-1, -6) and proteolytic enzymes and their inhibitors (MMPs, TIMPs) [19,20,21]. Our studies focused on the transcription factor Foxn1, whose deficiency in nude mice (Foxn1^−/−^) causes scar-less/scar-free healing [22,23]. It was revealed that Foxn1 regulates reparative (scar-forming) wound healing by engaging in re-epithelialization, epithelial-to-mesenchymal transition (EMT), and dWAT regulation [8]. Our recent data based on a mouse model (Foxn1^+/−^ and Foxn1^−/−^ mice) also showed that Foxn1 controls adipogenic signaling that regulates skin homeostasis and wound healing and affects susceptibility to diet-induced obesity [8,24]. 

Studies, for the most part, performed on animal models, particularly mice, do not always allow the data to be reflected in humans. For instance, the dWAT layer, which is separated from the fibroblast-rich dermis (reticular layer) by panniculus carnosus in rodents, is not definitely part of subcutaneous fat in humans [14]. Human dWAT, located below the reticular layer, which around hair follicles forms adipocyte-filled cone structures, has also been named “skin-associated fat” (SAF) and described as a body-wide depot distinguished from subcutaneous fat pads [9]. Furthermore, single-cell RNA sequencing analysis of mouse [25] and human [26] skin cells showed wide heterogeneity in dermal fibroblast populations but also differences in defined markers between murine vs. human skin. 

The complexity of human intact skin is also related to body regions, the age of individuals, and gender differences. In humans, men’s skin is thicker than women’s [27]. Age-related decreases in skin thickness start at the age of 50 in women and at the age of 20 in men [28,29]. Other skin parameters related to hydration, transepidermal water loss, sebum production, and microcirculation are higher in men, but skin pH is higher in women [30]. 

Although gender differences in human skin in the aspects of layer thickness, pH, hormones, and mechanical properties were broadly studied [27,28,30], there are very limited data considering how gender affects skin wound healing, particularly the composition/quality of post-wounded skin. 

The present study performed on human intact and scar skin focused on determining the gender differences in ECM composition, dWAT accumulation, and Foxn1 expression as a part of the skin response to injury. 

## 2. Materials and Methods

**Figure 1:**
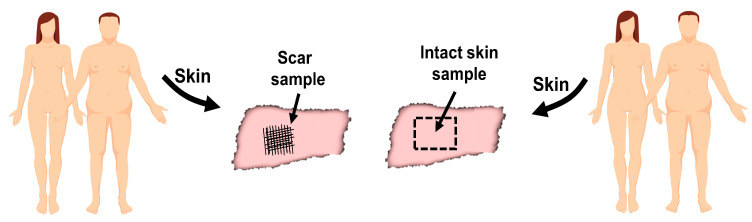
Scheme of human skin samples collection.

Skin tissues were obtained from surgically treated patients as intact skin and scar tissues (Figure 1). 

Immediately after skin collection in the surgery room, samples were carefully dissected, cut into three equal-sized specimens, and stored in liquid nitrogen (LN), followed by mRNA or protein isolation or cryostat sectioned histological examination. The number of human subjects comprised 39 patients, including 15 women aged 52–82 and 24 men aged 49–79. Data concerning patients are presented in Table 1.

This study was approved by the Bioethics Committee of the School of Medicine, University of Warmia and Mazury (Olsztyn, Poland), No. 28/2014. All human subjects gave written informed consent to donate discarded skin samples during surgery for the study. 

### 2.1. Isolation and Quantitative Real-Time PCR (qRT-PCR)

Frozen skin samples were powdered in liquid nitrogen using a prechilled mortar and pestle. Total RNA was extracted from skin samples using TRI Reagent (Sigma-Aldrich, St. Louis, MO, USA) according to the manufacturer’s instructions. The quality and quantity of isolated mRNA were estimated on NanoDrop 1000 (Thermo Fisher Scientific, Waltham, MA, USA) and through analysis of agarose gel after electrophoresis. Only samples displaying two clear bands of ribosomal RNA (18S rRNA and 28S rRNA) on the agarose gel were included for the following analyses. Genomic DNA was removed from RNA samples using a DNase I, Amplification Grade Kit (Invitrogen by Thermo Fisher Scientific). cDNA was synthesized from 500 ng of total RNA using the High-Capacity cDNA Reverse Transcription Kit with RNase Inhibitor (Applied Biosystems by Thermo Fisher Scientific). To measure the levels of *COLLAGEN 1A1* (Cat# Hs00164004_m1), *COLLAGEN 6A3* (Cat# Hs00915120_m1), *ELASTIN* (Cat# Hs00355783_m1), *FABP4* (Cat# Hs01086177_m1), *PPARγ* (Cat# Hs01115513_m1), *LEPTIN* (Cat# Hs00174877_m1), *FOXN1* (Cat# Hs00919268_m1), *GAPDH* (Cat# Hs02758991_g1) mRNA expression, Single Tube TaqMan Gene Expression Assays (Life Technologies by ThermoFisher Scientific) were used. Amplification was performed using the 7900HT Fast Real-Time PCR System under the following conditions: Initial denaturation for 10 min at 95 °C, followed by 40 cycles of 15 sec at 95 °C and 1 min at 60 °C. Each run included a standard curve based on aliquots of pooled skin RNA. All samples were analyzed in duplicates. mRNA expression levels were normalized to the reference gene *GAPDH* and multiplied by 10.

### 2.2. Protein Isolation and Western Blot Analysis

Frozen skin samples were powdered in liquid nitrogen using a prechilled mortar and pestle. For FOXN1 protein levels estimation, extraction of cytoplasmic and nuclear protein fractions was performed using NE-PER; Nuclear and Cytoplasmic Extraction Reagents (ThermoFisher Scientific), according to the manufacturer’s protocol. Protein concentration was measured by the infrared (IR)-based protein quantitation method with a Direct Detect Infrared Spectrometer (Merck, Darmstadt, Germany). Briefly, 35 μg of protein was separated on 9% Tricine gels and transferred to polyvinylidene difluoride membranes (Merck). The membranes were incubated with anti-FOXN1 (1:1000, Cat#113235 Abcam, Cambridge, MA, USA) or anti-β-actin (1:1000, Cat#8226, Abcam), followed by incubation with fluorescent secondary antibodies against Alexa Fluor 800 plus (1:10,000, Cat#A32735 ThermoFisher Scientific) or Cy5.5 (1:10,000, Cat#610-113-121, Rockland Immunochemicals, Inc. Limerick, PA, USA) for 1 h. Bands were visualized using the ChemiDoc Touch Imaging System (Bio-Rad Laboratories, Inc., Hercules, CA, USA) and analyzed using Image Lab Software version 6.0.1 (Bio-Rad Laboratories, Inc.) according to the manufacturer’s protocol.

### 2.3. Histology

Analyses were performed on 8 μm cryostat sections fixed in ice-cold methanol/acetone (1:1) for 20 min. Skin tissues were stained with Harris’ hematoxylin solution (Harris Modified HHS32-1L, Sigma-Aldrich, St. Louis, MO, USA ) for 1 min, then rinsed in tap water until the water was colorless. Next, staining was performed with eosin Y ethanol solution (Eozin Y solution, alcoholic HT110132-1L, Sigma-Aldrich Co.) for 30 s and quickly rinsed in distilled water.

Immunohistochemical and immunofluorescent analyses were performed using the protocols described previously [8,23]. For immunofluorescent procedures, the following primary antibodies were used: aSMA (1:200, Cat# RB9010-P, Thermo Fisher Scientific), E-cadherin (1:1000, Cat# 13-1900, Thermo Fisher Scientific), Vimentin (1:300, Cat# 92547, Abcam), Keratin 10 (1:1500, Cat# PRB159P-100, Covance, Princeton, NJ, USA), Involucrin (1:1500, Cat# PRB 140C-200, Covance), and FSP1 (1:800, Cat#27957, Abcam). The following secondary antibodies were used: Alexa Fluor 594 (1:200, Cat# A11037, Invitrogen) or Alexa Fluor 488 (1:200, Cat# A 21206, Invitrogen, Thermo Fisher Scientific, Waltham, MA, USA). Nuclei were counterstained with ProLongVR Gold Antifade Mountant with DAPI (Life Technologies, Thermo Fisher Scientific, Waltham, MA, USA).

Immunohistochemical staining for the presence of α-SMA and Perlipin-1 was performed with anti-α-SMA (1:200, Cat# RB9010-P, ThermoFisher Scientific) or anti-perlipin-1 (1:200, Cat#3526, Abcam). Antibody binding was detected with the ABC complex (Vectastain ABC Kit, Vector Laboratories, Inc., Burlingame, CA, USA). In control sections, primary antibodies were substituted with non-specific-immunoglobulin G (IgG). Peroxidase activity was revealed using 3.30-diaminobenzidine (Sigma-Aldrich, St. Louis, MO, USA) as a substrate. Sections were counterstained with hematoxylin. 

All slides were visualized using an Olympus microscope (BX43), photographed with an Olympus digital camera (XC50), and analyzed with Olympus CellSens Software version Standard 2.2 (Olympus, Tokyo, Japan).

Slides for collagen networks (collagen 1 and collagen 3) were stained with picrosirius red (Direct Red 80, Catalog# 36-554-8, Sigma-Aldrich Co.) for 1 h. After staining, sections were washed in two changes of acidified water (0.5% acetic acid) and dehydrated with three changes of 100% ethanol. The sections were visualized using: (i) A GRUNDIUM OCUS^®^ microscope slide scanner; (ii) an Olympus microscope (BX43) with polarized light; and photographed with an Olympus digital camera (XC50). Analysis of collagen fibers: yellow-red (collagen type I) and green (collagen type III) was performed as a relative object count [%] with Olympus CellSens Dimension Software (Olympus, Tokyo, Japan).

### 2.4. Statistical Analyses

Statistical analyses were performed using the GraphPad PRISM, version 6.02 software (GraphPad Software Inc., La Jolla, CA, USA). The significance of differences between groups was assessed using one-way analysis of variance with Tukey’s test for multiple comparisons. Data are expressed as the mean ± standard error; * *p* < 0.05, ** *p* < 0.01, *** *p* < 0.001.

## 3. Results

### 3.1. Human Skin Histological Differences: Abdomen vs. Thigh

**Figure 2:**
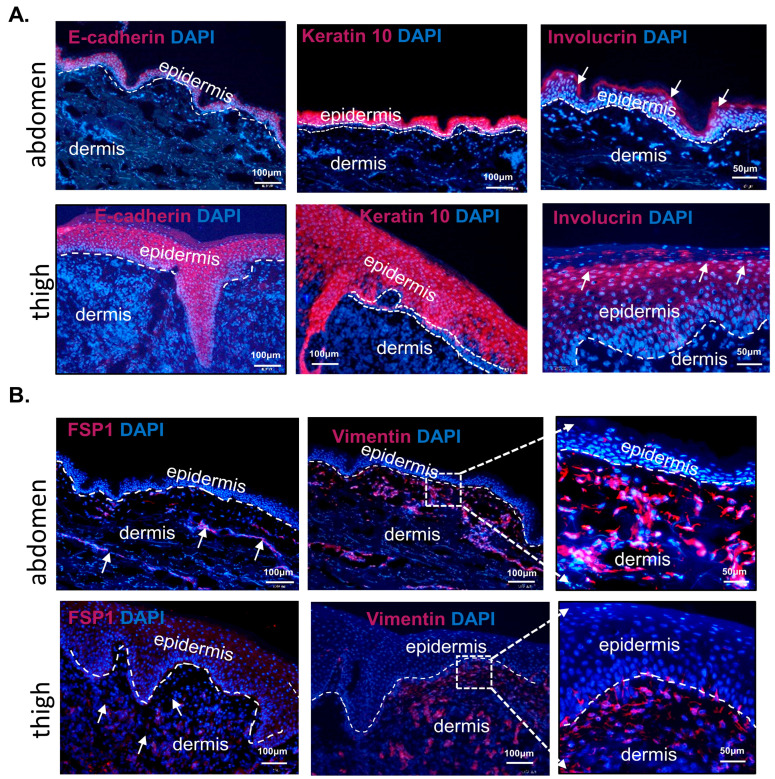
Immunofluorescent localization of (**A**) epidermal and (**B**) dermal markers in human skin from thigh or abdomen. Arrows indicate product of immunofluorescent reaction.

Skin anatomy, histomorphology, and even the immunological barrier show alterations related to body regions and individual age [31,32]. For the purpose of our study, we initially collected skin from two regions of the body: The thigh and abdomen. To characterize potential similarities or differences between these two skin areas, we performed immunofluorescent detection of epidermal markers: E-cadherin, keratin 10, and involucrin (Figure 2A) and dermal markers: Vimentin and fibroblast-specific protein 1 (FSP1) (Figure 2B). E-cadherin, the global marker of epithelium, is localized to all layers of the epidermis. Keratin 10 is a marker of differentiated keratinocytes confined to all epidermal layers but not to the basal one (Figure 2A) of the thigh and abdomen. Involucrin was detected particularly in keratinocytes of cornified envelopes (Figure 2A). No differences were observed in epidermal marker localization between skin samples from the abdomen or thigh (Figure 2A). In contrast, markers of dermal fibroblasts, FSP1 and vimentin, showed different immunolocalization between the thigh and abdomen (Figure 2B), with a denser accumulation of the product of the FSP1 and vimentin immunoreaction in the abdominal region than in the thigh dermis (Figure 2B). Due to detected differences in dermal markers but also considering the higher prevalence of abdominal surgery compared to thigh, for our next examination, we focused on abdomen skin samples. 

### 3.2. Extracellular Matrix Composition in Intact and Scar Skin: Females vs. Males

**Figure 3:**
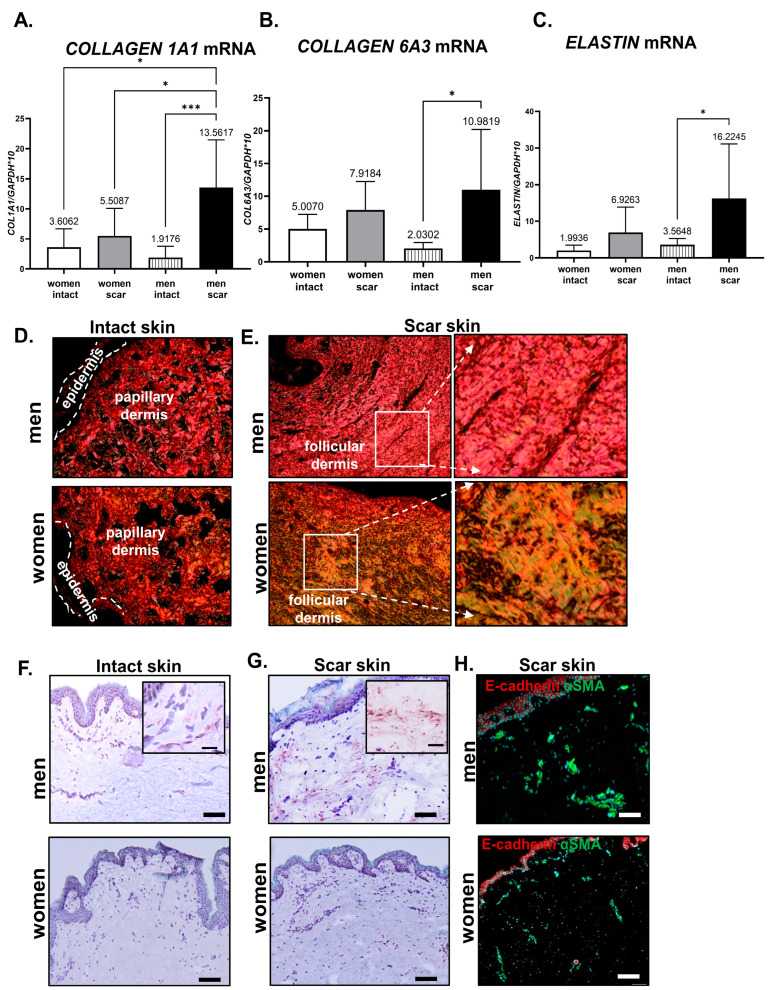
Extracellular matrix (ECM) components in intact or scar tissues collected from human abdomen skin. qRT-PCR of *COLLAGEN 1A1* (**A**), *COLLAGEN 6A3* (**B**), and *ELASTIN* (**C**) mRNA expression analysis. Representative histological sections of human skin collected from: intact (**D**) or scar (**E**) tissues stained with picrosirius red. Collagen fibers: yellow—red (collagen type I) and green (collagen type III). Immunohistochemical (**F**,**G**) and immunofluorescent (**H**) localization of αSMA in intact (**F**) or scar (**G**,**H**) human skin. Scar bar: 100 µm (**F**,**G**), 50 µm (**H**), and insets. Data (**A**–**C**) represents mean ± SD; *p* < 0.05 (*); *p* < 0.001 (***).

To quantitatively analyze the impact of gender on intact and scar skin, the mRNA expression levels of the ECM components: *COLLAGEN 1A1* (Figure 3A), *COLLAGEN 6A3* (Figure 3B), and *ELASTIN* (Figure 3C) were performed. Collagen I and III as the “skeleton” of connective tissue and collagen VI as an interstitial matrix assembly element [33] are the major components of the skin ECM, for which restoration is required in the post-injured area. The expression levels of *COLLAGEN 1A1* (Figure 3A) and *COLLAGEN 6A3* (Figure 3B) were higher in scar than in intact skin samples from men and women, although a statistically significant increase was observed exclusively for men. The *COLLAGEN 1A1* mRNA in intact skin showed similar levels of expression between men and women (Figure 3A). However, scar tissues displayed large differences in *COLLAGEN 1A1* mRNA levels that were extremely high for men with respect to the intact skin of men (*p* < 0.001) or scar (*p* < 0.05) skin of women (Figure 3A)*. COLLAGEN 6A3* also displayed high levels of mRNA expression in the scar skin of men in comparison to intact skin (*p* < 0.05), but no differences were observed in relation to the skin of women: Intact or scar (Figure 3B). To further characterize the collagen content in situ, histological skin sections were stained with picrosirius red and analyzed under polarized light microscopy [34] (Figure 3D,E). 

Picrosirius red staining marks the collagen bundles in red-yellow that represent collagen type I, and green staining represents collagen type III fibers on a black background of sections (Figure 3D,E). The intact skin of men and women did not differ and showed basketweave-like pattern collagen bundles abundant in collagen I (red color) (Figure 3D). The differences were detected in scar samples. The scar skin of men is characterized by thick collagen fibers (red color indicating collagen I) in parallel arrangements that are characteristic of scars (Figure 3E, upper panel). Interestingly, the scar skin of women displayed thick collagen fiber accumulation in the injury area, but with a dominant color of green/yellow, which indicates the accumulation of collagen III (Figure 3E, lower panel). 

Next, we examined the *ELASTIN* mRNA levels as an ECM component and found that production is absent in the mature state of the skin, but synthesis is induced by injury [35]. Indeed, the intact skin of both men and women displayed similar and very low levels of *ELASTIN* mRNA (Figure 3C). The increase in *ELASTIN* transcript levels was detected in scars compared to the healthy skin of men (*p* < 0.05; Figure 3C). Myofibroblasts, the cells that rapidly accumulate in post-injured skin, displayed enhanced synthetic activity of ECM proteins. We used the immunohistochemistry technique (Figure 3F,G) and immunofluorescent (Figure 3H) detection assays for α-SMA as a marker of myofibroblasts. Intact skin showed no (women) or very few cells (men) positive for α-SMA (Figure 3F). The increase in α-SMA presence was detected in women’s and men’s scar skin (Figure 3G,H). Particularly, scar skin sections collected from men showed high immunoreactivity for α-SMA observed as an immunohistochemical product of reaction that was confirmed by an immunofluorescent detection assay (Figure 3G,H). 

Collectively, the analysis of ECM components showed gender differences in ECM reestablishment in scar skin. Post-wounded skin of men showed highly increased levels of: *COLLAGEN 1A1*, *COLLAGEN 6A3*, and *ELASTIN* mRNA expression, the accumulation of thick collagen I-positive fibers, and the accumulation of α-SMA-positive cells. On the contrary, post-injured skin of women displayed a slight increase (in comparison to intact skin of women and scar men skin) in the expression of ECM components and an increase in collagen III accumulation in the area of injured skin.

### 3.3. Differences in Skin Adipogenic Propensities between Men and Women

**Figure 4:**
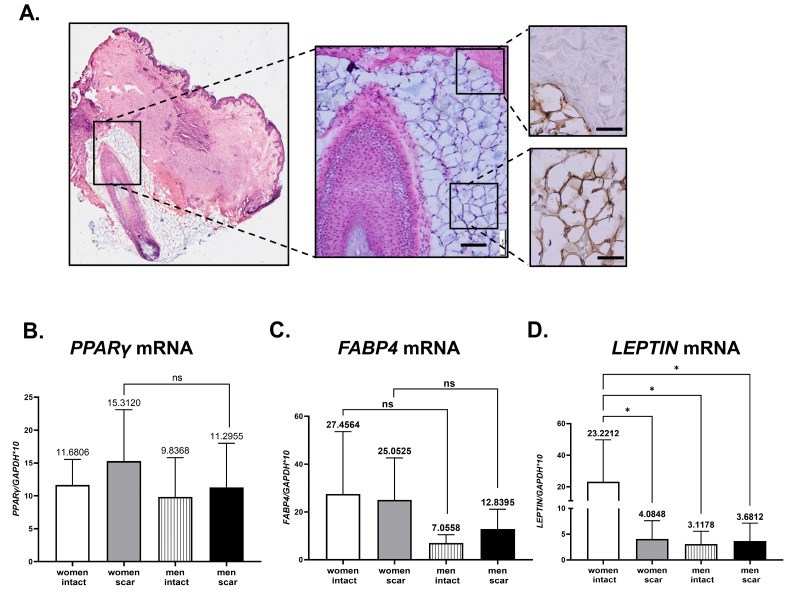
Human skin adipogenic propensities. (**A**) Representative histological section of human intact skin stained with hematoxylin-eosin or immunostained for perilipin-1 positivity (insets). qRT-PCR of *PPARγ* (**B**), *FABP4* (**C**), and *LEPTIN* (**D**) mRNA expression analysis in intact and scar skin tissues from men or women. Scar bar 100 µm (**A**), 50 µm in insets. Data represents mean ± SD; *p* < 0.05 (*).

Dermal white adipose tissue (dWAT), originally defined in mouse skin [36], in human skin is not clearly demarcated from other dermal components and have also been called skin-associated fat (SAF) [9]. Consistent with the unique cone geometry of human dWAT [37], the histological analysis of abdominal human skin revealed dermal adipocytes surrounding the hair follicle in the form of a dermal cone (Figure 4A). To confirm the adipocyte characteristic of cells, immunohistochemical detection of perilipin-1 (adipocyte-specific marker) revealed a positive reaction confined to a narrow line of lipid layer surrounding the centrally allocated lipid droplet (Figure 4A, insets).

Next, using the same set of samples as for analyses of ECM components (see Figure 3A–C), we evaluated the levels of mRNA expression for key factors of adipogenesis: *PPARγ* (Figure 4B), *FABP4* (Figure 4C), and *LEPTIN* (Figure 4D). Generally, we observed a tendency towards higher levels of adipogenic-related genes in skin samples from women than men, regardless of intact or post-injured/scar skin (Figure 4B–D). However, statistically significant differences were detected for *LEPTIN* mRNA, where women with intact skin showed six-time higher levels of expression in comparison to women with scar (*p* < 0.05) or men with intact (*p* < 0.05) skin (Figure 4D).

Overall, the adipogenic potential analyses showed that women’s skin displayed a tendency toward increased adipose propensities in comparison to men’s skin.

### 3.4. Transcription Factor FOXN1 in Human Skin

**Figure 5:**
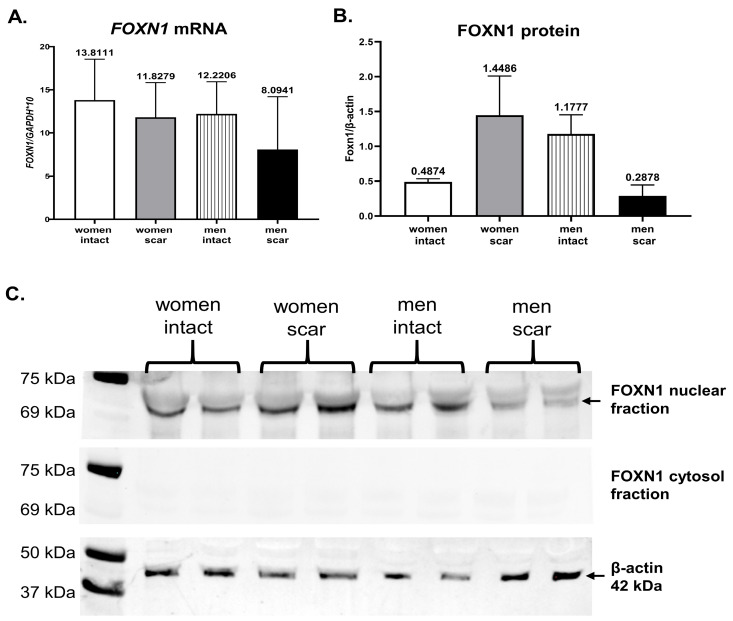
FOXN1 in human abdominal intact or scar skin. qRT-PCR of FOXN1 mRNA (**A**) and protein by densitometric (**B**) and Western blot (**C**) analyses.

Our previous data based on a mouse model revealed that the transcription factor Foxn1 expressed in the epidermis regulates skin wound healing and initiates the cascade of adipogenic signaling in mouse skin [8,23]. To assess FOXN1 expression in human skin qRT-PCR (Figure 5A), Western blot and densitometry (Figure 5B,C) analyses were performed. Human skin showed no differences in *FOXN1* mRNA expression levels between men and women, regardless of intact or scar samples (Figure 5A). The data was supported by Western blot FOXN1 protein analysis (Figure 5B,C). For FOXN1 protein detection, we used separate nuclear and cytosol skin sample fractions. The cytosol fraction showed no FOXN1 protein presence but clear bands of housekeeping β-actin protein, even in size, for all skin samples (Figure 5C). The nuclear fraction displayed well-defined FOXN1 protein bands (Figure 5C). Highly abundant protein bands were observed in post-injured women’s skin, with a diminished reaction in men’s scar skin (Figure 5B,C). 

## 4. Discussion

The present study demonstrated gender differences in human skin, particularly in post-injured/scar tissues. Skin scar tissues of men showed higher than women expression of *COLLAGEN I*, *COLLAGEN VI*, and *ELASTIN* mRNA and denser accumulation of αSMA-positive cells. Post-injured women’s skin is characterized by an accumulation of *COLLAGEN III* that is hardly present in men’s scar tissues. The analysis of skin adipogenic-related genes showed higher levels of mRNA expression for *PPARγ*, *FABP4*, and *LEPTIN* in women than men, particularly in intact skin. 

In the skin as well as in other tissues, the ECM forms not only structure elements but also functions as a bioactive milieu, facilitating and actively participating in cell signaling exchange. Collagen is the main component of the ECM, and its rate of synthesis/degradation, composition, and architecture define normal skin vs. normotrophic, hypertrophic, or keloid scars [38]. Regardless of the type of scar, collagen synthesis increases in the scar as compared to normal skin [39,40]. Our data are in agreement with previous studies showing an increase in *COLLAGEN I* and *COLLAGEN VI* expression in scar tissues. However, we also detected that gender affects *COLLAGEN* expression, largely increasing its mRNA levels in the scar tissues of men, with a slight increase in women’s scars. Likewise, the increase in *ELASTIN* expression was predominantly detected in men’s scars, which is in agreement with reports that elastin production is absent in mature skin but synthesis is induced by injury [35].

The large population study showed and confirmed preceding data related to collagen fiber arrangement and architecture in intact and scar skin [38]. Normal/intact skin showed a basketweave pattern of collagen bundle arrangement, whereas scar collagen bundles are oriented parallel to the epithelial surface [38]. To estimate collagen arrangement in our study, we applied Picrosirius red staining, which showed the accumulation of parallel-oriented collagen bundles in scar tissues but also allowed us to distinguish between two types of collagen: Collagen I and collagen III. Collagen I builds up the intact skin, and it is the major ECM element of post-wounded skin. Collagen III is synthesized during normal/reparative skin healing. However, collagen III is replaced by collagen I at the remodeling (final stage) of wound healing, which for humans on average occurs 6–12 months after injury [41]. The accumulation and sustained content of collagen III characterize post-injured skin, which is predisposed to regenerative healing, as was shown for mammalian fetuses [42]. High levels of collagen III expression were also detected in dermal fibroblasts of Foxn1^−/−^ mice, which heal skin injuries in a regenerative manner [22,23]. 

Higher than in normal/mature scars, collagen III content was detected in human hypertrophic scars, particularly in the papillary dermis [43,44]. High and prolonged levels of collagen III in hypertrophic scars have been attributed to the continued rapid turnover of collagen, indicating the characteristics of embryonic collagen [45]. Furthermore, in humans, mutations in the pro-collagen III gene cause Ehlers–Danlos syndrome IV, which is characterized by wounds with an abnormally long scarring process and enlargement of scars [46].

Whether detected in our study, sustained collagen III content in women’s scars indicates propensities to regenerative/embryonic skin wound healing and requires further study. Interestingly, single-cell RNA-seq analysis of human dermal cells revealed sex-related differences, particularly within matrix-associated genes: VIM, MFAP4, and FBLN1, which were higher in fibroblasts collected from women than in men’s skin [26]. Single-cell RNA-seq has also been applied to analyze differences between fibroblasts of healthy skin and hypertrophic scars in humans [47]. Among the up-regulated genes in the hypertrophic set of fibroblasts, there are those functionally related to collagen and ECM-modified genes: BGN, COL14A1, COL1A1/2, COL3A1, COL5A1/2, FN1, MMP23B, OGN, and PCOLCE [47,48].

dWAT, the newly defined layer within the dermis, has gained a lot of attention due to its distinct from other fat depots (i.e., subcutaneous, intraperitoneal, or gonadal), developmental and functional characteristics, and function [36,49,50]. In mice, the age- and stage-dependent modulation of hair follicle cycle-dependent modulation of dWAT has been broadly studied [12,51]. The reduction in the dWAT layer is associated with aging, as was observed in different knock-out mouse models [52]. However, HFD can rescue age-related dWAT reduction that reverses dermis thickness to that observed for middle-aged mice [17]. Moreover, the size and thickness of dWAT undergo changes along with the hair cycle, being the largest in anagen and reduced in catagen [12].

A study by Azzi et al. on a skin mouse model revealed sexual dimorphism in dWAT content, showing about an 11-fold thicker layer of dWAT (called in the study hypodermis) in females than in males [53]. Moreover, steroid hormone treatment altered dWAT to decrease thickness through dihydrotestosterone (DHT), estradiol (E2), or dehydroepiandrosterone (DHEA) treatment, whereas the dWAT increase was observed in gonadectomized (GDX) male and female mice [53]. A recent human study by Kasza et al., 2021 analyzing SAF revealed that it is much thicker in women (by 60% on average) than in men [9]. Moreover, human SAF depot, in contrast to mouse dWAT, becomes lipolytic, generating heat in response to β-adrenergic stimulation. The authors conclude that in humans, SAF provides alternatives to BAT (brown adipose tissue) in heat production [9]. The data from our study support human gender differences in dWAT/ SAF. Higher levels of adipogenic-related gene expression were detected in intact female skin than in male skin. Particularly, the high levels of *LEPTIN* mRNA indicate not only the adipogenic potential, as *PPARγ* and *FABP4* mRNA expression levels imply, but also the features of mature adipocytes with the ability to produce and release leptin [54]. The expression of adipogenic-related genes seems to correspond to FOXN1 expression in human skin (compare Figure 4 and Figure 5). Higher levels of FOXN1 mRNA and protein were detected in women than in men’s scar skin. Interestingly, our previous study performed on a mouse model showed that Foxn1 acts as a pro-scarring factor in the skin wound healing process but also plays a stimulatory role in skin adipogenesis [8,23]. Furthermore, our recent data showed that the introduction of Foxn1 into the post-injured skin of Foxn1^−/−^ mice increased the mRNA expression of *Pparγ*, *Glut 4*, and *Fasn* but also the mRNA of *Igf2*, the growth factor participating in adipogenic signal transduction [24]. Those data correspond with the present human study implying the stimulatory role of Foxn1 in post-injured dWAT reestablishment. Whether the gender differences in FOXN1 in human skin shown in the present data may be attributable to increased adipogenic propensities in women’s skin merits future studies. 

Human gender differences in skin architecture, anatomy, and physiology have been broadly studied (reviewed in Tur, E. 1997 [28]. Men have thicker skin than women across all ages, but they also have slower skin wound healing rates than women [55]. 

The sex-related differences in anatomy, physiology, and epidemiology also reflect the manifestation of several skin-related diseases. Women are more prone to autoimmune and allergic diseases, whereas men are more predisposed to atopic dermatitis and severe psoriasis [27,30,56,57]. Since skin acts as a steroidogenic organ that produces, metabolizes, and responds to sex hormones [27,58] the observed/detected gender-related differences are attributable to sex hormones, as was suggested previously [27,30,56,57]. It was supported by observations of an increase in skin thickness in post-menopausal women following treatment with conjugated estrogens [28]. Moreover, the reduction of the ratio between collagen III and collagen I in menopausal women showed an increase after hormone replacement therapy [28]. Furthermore, topical estrogen application diminished age-related delays in wound healing for men and women [59]. 

In conclusion, our data confirm and extend (dWAT layer) the differences between men and women in intact skin but also indicate gender differences in human scar tissues. This knowledge in the future may contribute to the more effective and gender-tailored improvement of skin care interventions, particularly for abnormal scar formation. 

## Figures and Tables

**Table 1:** Subjects’ characteristics.

Abdominal Skin Samples	Sex	Age	MEAN AGE	Number of Subjects (n)
Intact skin	Female	57–82	65.5	6
Scar	Female	52–75	60	9
Intact skin	Male	49–77	63.4	12
Scar	Male	52–79	65.6	12

## Data Availability

Not applicable.
